# The impact of sarcopenia on nutritional status in elderly patients with gastrointestinal tumors

**DOI:** 10.1038/s41598-023-37470-w

**Published:** 2023-06-26

**Authors:** Dejie Ai, Ningrong Ding, Hui Wu

**Affiliations:** 1 Department of Nutrition, Liaoning Cancer Hospital, Shenyang, 110801 China; 2 Research Center, Shanghai Healink Medical Information Consulting Co., Ltd, No. 258 Pingyang Road, Minhang District, Shanghai, 201102 China

**Keywords:** Gastrointestinal diseases, Gastroenterology, Oncology

## Abstract

This study aimed to analyze the impact of sarcopenia on nutritional status in elderly patients with gastrointestinal tumors. We conducted a study of 146 elderly patients with gastrointestinal tumors in our hospital from January 2020 to June 2022. Patients enrolled were divided into normal nutritional status group (80 patients) and high nutritional risk group (66 patients) according to their nutritional status. The clinical information and nutritional status of the two groups were compared and analyzed. Multivariate logistic regression was done to analyze the risk factors of nutritional status in elderly patients with gastrointestinal tumors; and receiver operating characteristic (ROC) curve was used to evaluate the predictive value of sarcopenia on nutritional status in elderly patients with gastrointestinal tumors. Malnutrition was present in 66 (45.21%) of 146 elderly patients with gastrointestinal cancer. There was no significant difference in gender, age, and tumor location between the two groups (*P* > 0.05). However, statistically significant difference was observed between the two groups in terms of BMI, tumor staging, calf circumference, the third lumbar vertebra skeletal muscle index (L3-SMI), muscle strength, 6 m walking speed, short physical performance battery (SPPB) score, PG-SGA score, and sarcopenia (*p* < 0.05). The independent variables were set as BMI (≤ 21.27 kg/cm^2^), tumor staging (stage II to III), calf circumference (male: ≤ 30.01 cm, female: ≤ 29.37 cm), L3-SMI (male: ≤ 41.29 cm^2^/m^2^, female: ≤ 34.29 cm^2^/m^2^), muscle strength (male: ≤ 22.32 kg, female: ≤ 16.39 kg), 6 m walking speed (≤ 0.89 m/s), SPPB score (≤ 8.67 points), PG-SGA score (> 3 points), and sarcopenia. The dependent variable was malnutrition in elderly patients with gastrointestinal tumors. A univariate logistic regression analysis was conducted, showing that the influencing factors for malnutrition in elderly patients with gastrointestinal tumors were L3-SMI (male: ≤ 41.29 cm^2^/m^2^; female: ≤ 34.29 cm^2^/m^2^), muscle strength (male: ≤ 22.32 kg; female: ≤ 16.39 kg), BMI (≤ 21.27 kg/cm^2^), SPPB score (≤ 8.67 points), PG-SGA score (> 3 points) and sarcopenia. Moreover, the independent variables were set as L3-SMI (male: ≤ 41.29 cm^2^/m^2^; female: ≤ 34.29 cm^2^/m^2^), muscle strength (male: ≤ 22.32 kg; female: ≤ 16.39 kg), BMI (≤ 21.27 kg/cm^2^), SPPB score (≤ 8.67 points), PG-SGA score (> 3 points), and sarcopenia. The dependent variable was malnutrition in elderly patients with gastrointestinal tumors. The multivariate logistic regression analysis revealed that the influencing factors of malnutrition in elderly patients with gastrointestinal tumors were BMI (≤ 21.27 kg/cm^2^) and sarcopenia. The ROC curve of BMI (≤ 21.27 kg/cm^2^) and sarcopenia, and the area under the curve (AUC) of BMI (≤ 21.27 kg/cm^2^) and sarcopenia predicting malnutrition in elderly gastrointestinal cancer patients were 0.681 and 0.881, respectively. The influencing factors of malnutrition in elderly patients with gastrointestinal tumors were BMI (≤ 21.27 kg/cm^2^) and sarcopenia, and the two factors may exert predictive value in terms of the occurrence of malnutrition in such patients.

## Introduction

The incidence of malnutrition among patients afflicted with malignant tumors varies between 40 and 80%, with the most elevated incidence of nutritional vulnerability observed in patients with gastrointestinal tumors, with a range of 84.3–90.0%^1,2^. China is regarded as one of the countries with a high incidence of gastrointestinal malignant tumors, and the elderly are the main population of such condition. Patients with gastrointestinal tumors generally present with high nutritional risks and sarcopenia due to gastrointestinal dysfunction and tumor progression, and the condition can be more serious for elderly patients^3^. Sarcopenia is an acute or chronic syndrome of decreased skeletal muscle content associated with age, accompanied by reduced muscle strength and physical function^4^. Earlier studies are available to show that, approximately 30% to 40% of patients with malignant tumors may develop sarcopenia that was considered a poor prognostic factor for such patients^5,6^. Elderly individuals diagnosed with gastrointestinal tumors frequently experience symptoms such as loss of appetite and malnutrition, which can disturb the equilibrium of protein assimilation and breakdown. This disturbance can result in a state of heightened catabolism and reduced anabolism, ultimately culminating in the onset of sarcopenia^7^. In addition, elderly patients with gastrointestinal tumors frequently present with malabsorption of digestive tract, which renders them more susceptible to sarcopenia^8^. However, there is a scarcity of studies on the relationship between sarcopenia and nutritional status of elderly gastrointestinal cancer patients. Herein, our study was conducted to explore the impact of sarcopenia on nutritional status of elderly gastrointestinal cancer patients, and in order to provide a theoretical framework for the clinical subunits domain of gastrointestinal tumor complicated with sarcopenia.

## Patients and methods

### Participants

We included a total of 146 elderly patients with gastrointestinal tumors in our hospital from January 2020 and June 2022. Patients enrolled were divided into normal nutritional status group (80 patients) and high nutritional risk group (66 patients) according to their nutritional status. There were 42 males and 38 females in the normal nutritional status group, with age ranged from 61 to 86 years old (mean: 71.29 ± 10.37). There were 36 males and 30 females in the elderly gastrointestinal tumor group, with age ranged from 61 to 86 years (mean: 71.03 ± 10.76).

The study was approved by the Ethics Committee of Liaoning Cancer Hospital (No. LNEIC-076). The formulation of this research protocol was conducted in accordance with the relevant requirements of the World Medical Association Helsinki Declaration. All patients have signed an informed consent form.

According to the formula: nc = (μ1 − α/2 + μ1 − β)^2*s^2(1 + 1/k)/(μt − μc)^2 = 2 × (1.96 + 0.84)^2 × 0.86^2/(2.48–2.05)^2 = 50^9^, the minimum number of cases in each group was not less than 50. The final number of cases collected from the hospital were 80 elderly gastrointestinal tumors patients and 66 elderly patients with gastrointestinal tumors combined with malnutrition.

### Selection criteria

#### Inclusion criteria

1. Patients with gastrointestinal tumors confirmed by histopathology;
2. Complete information of the disease;
3. Patients receiving anti-tumor treatment and surgical treatment before participating our study;
4. Patients with clear awareness, autonomous ability, and willingness to cooperate in completing examinations, assessments, and physical measurements.

### Exclusion criteria

1. Patients with implanted devices such as fixed steel plates, cardiac stents, and pacemakers installed in the body;
2. Patients with severe attenuation of important organs including heart, lung, liver, kidney, etc.;
3. Patients with severe edema and ascites;
4. Patients complicated with chronic obstructive pulmonary disease, diabetes, cardiac and renal insufficiency and other basic diseases that may cause muscle decline;
5. Patients who were unable to complete grip strength and physical fitness tests;
6. Patients who was unable to undergo CT examination.

## Methods

### Clinical information

The clinical information included gender, age, body mass index (BMI), medical history, pathological diagnosis, tumor staging, abdominal CT examination, etc.

### Nutrition risk screening

The Patient-Generated Subjective Global Assessment (PG-SGA)^10^ recommended by the American of Society for Nutrition, and the Chinese Anti-Cancer Association of Nutrition and Cancer Treatment Support Committee was used to assess the nutritional status of patients within 48 h of admission. Nutrition assessment includes patient self-assessment (body mass, eating status, symptoms, and physical activity) and medical staff assessment (disease, stress status, physical examination). PG-SGA scores of 0–2 indicates normal nutrition, ≥ 3 malnutrition.

### Diagnosis of sarcopenia

Brazilian scholar Barbosa Silva proposed a screening method formed in 2016 by combining the Strength, Assistance in walking, Rise from a chair, Climb stairs, Falls history (SARC-F) questionnaire^11^ with calf circumference indicators. The SARC-F is a 5-item questionnaire. The scores range from 0 to 10, with 0 to 2 points for each item. When the calf circumference was less than the critical value (male ≤ 34 cm; female ≤ 33 cm), a score of 10 points was recorded, with a total score of 0–20 points. A total score of ≥ 11 points indicated there was risk of sarcopenia. The researchers diagnosed patients with positive SARC-CalF scales according to the criteria of the Asian Working Group for Sarcopenia (AWGS)^12^, and comprehensively evaluate them from three aspects: (1) The muscle quality evaluation was performed based on the CT imaging of the third lumbar vertebra skeletal muscle index (L3-SMI) to reflect the skeletal muscle volume, L3SMI = the total area of the third lumbar vertebra skeletal muscle/square of height, and the cutoff value of muscle mass reduction was indicated by the use of L3-SMI < 52.4 cm^2^/m^2^ for males and L3-SMI < 38.5 cm^2^/m^2^ for females; (2) The muscle strength was measured based on the grip strength, using a spring grip in the standing position, and using the dominant hand or both hands to contract equally with the maximum force for elbow extension. At least two tests were conducted, and the maximum value was taken. A decrease in muscle strength was judged when that value was < 28.0 kg for males, and < 18.0 kg for females; (3) Decreased somatic function was determined when the 6 m-walking speed was < 1.0 m/s; (4) The short physical performance battery (SPPB)^13^ established by the National Institute on Aging, part of the National Institutes of Health, was used to evaluate the physical activity ability of patients, with a total of three subtests that combines the results of the standing balance, 2.44 m gait speed, and five sit-to-stand. The measurement was repeated twice for each test, and the shortest time value was recorded. Each subtest has a score of 4 points, with a full score of 12 points. Based on the 2019 consensus released by the AWGS, SPPB ≤ 9 points indicates decreased physical function. This study was conducted based on the diagnostic criteria of AWGS, where sarcopenia could be diagnosed with the decreased muscle mass and muscle strength or body function. The abovementioned evaluation and measurement were completed within 24–48 h of the admission of patients.

Prior to commencing the investigation, a training manual was disseminated to furnish investigators with consistent and standardized professional training in the collection of clinical data, nutritional risk screening, and diagnostic criteria for sarcopenia. It was mandated that participation in the investigation was contingent upon successfully passing the training examination. During the survey, uniform explanatory language was used to explain and answer questions to the participants. After signing the informed consent form, the participants filled in the questionnaire by themselves that was further collected by studiers on the spot. For those who were unable to fill in the questionnaire themselves or had difficulty filling in the questionnaire, the investigator would ask and fill in the answers on their behalf.

### Observation indicators

The clinical data and the nutritional status of the two groups was compared and analyzed. Multivariate logistic regression was done to analyze the risk factors of nutritional status in elderly patients with gastrointestinal tumors; and receiver operating characteristic (ROC) curve was used to evaluate the predictive value of sarcopenia on nutritional status in elderly patients with gastrointestinal tumors.

### Statistical analysis

SPSS 21.0 software (IBM Corp., Armonk, NY, USA) and Excel were applied to analyze the data and establish a database. The measurement data conforming to the normal distribution were expressed in $\overline{x}$ ± s, and the overall comparison of data in each group was performed using one-way analysis of variance. Pairwise comparisons of data between groups and within groups were performed using the LSD method. The counting data was expressed in percentage (%) and compared using chi-square χ^2^ test. Multivariate logistic regression was done to analyze the risk factors of nutritional status in elderly patients with gastrointestinal cancer, and ROC curve was used to evaluate the predictive value of sarcopenia in elderly patients with gastrointestinal cancer, A *P* < 0.05 represented significant difference.

### Informed consent

Informed consent was obtained from all participants included in the study.

## Results

### Comparison of general information

In our study, malnutrition was present in 66 (45.21%) of 146 elderly patients with gastrointestinal cancer. There was no significant difference in gender, age, and tumor location between the two groups (*p* > 0.05). However, statistically significant difference was observed between the two groups in terms of BMI, tumor staging, calf circumference, L3-SMI, muscle strength, 6 m walking speed, SPPB score, PG-SGA score, and sarcopenia (*p* < 0.05), as shown in Table 1.

**Table 1:** Comparison of general information.

Indicator	Normal nutritional status group (n = 80)	High nutritional risk group (n = 66)	*χ*^*2*^*/t*	*p*
Gender (male/female)(n)	42/38	36/30	0.061	0.085
Age (year)	71.29 ± 10.37	71.03 ± 10.76	0.148	0.883
BMI (kg/cm^2^)	23.09 ± 4.09	21.27 ± 4.23	2.635	0.009
Tumor location (n)			0.947	0.918
Gastric cancer	29	20		
Liver cancer	7	6		
Pancreatic cancer	12	11		
Rectal cancer	19	15		
Colon cancer	13	14		
Tumor staging (n)			24.429	< 0.001
Stage I	12	0		
Stage II	53	32		
Stage III	15	34		
Calf circumference (cm)				
Male	32.67 ± 3.01	30.01 ± 3.23	5.141	< 0.001
Female	31.38 ± 3.28	29.37 ± 3.45	3.582	< 0.001
L3-SMI (cm^2^/m^2^)				
Male	56.38 ± 6.19	41.29 ± 6.08	14.778	< 0.001
Female	40.76 ± 6.12	34.29 ± 6.36		< 0.001
Muscle/grip strength (kg)				
Male	29.76 ± 2.86	22.32 ± 2.81	6.246	< 0.001
Female	20.03 ± 2.67	16.39 ± 2.31	8.709	< 0.001
6 m walking speed (m/s)	1.13 ± 0.27	0.89 ± 0.21	5.897	< 0.001
SPPB score (point)	9.82 ± 1.02	8.67 ± 1.03	6.744	< 0.001
PG-SGA score (point)			146.00	< 0.001
< 3 points	80	0		
≥ 3 points	0	66		
Sarcopenia (n)	1	20	24.788	< 0.001
Complication (n)				
Hypertension	8	6	0.034	0.853
COPD	10	9	0.041	0.839
Tobacco use (n)				
Alcohol use (n)				

*SPPB* short physical performance battery, *PG-SGA* patient-generated subjective global assessment, *COPD* chronic obstructive pulmonary diseases.

### Univariate logistic regression analysis of the influencing factors of malnutrition in elderly patients with gastrointestinal tumors

The independent variables were set as BMI (≤ 21.27 kg/cm^2^), tumor staging (stage II to III), calf circumference (male: ≤ 30.01 cm, female: ≤ 29.37 cm), L3-SMI (male: ≤ 41.29 cm^2^/m^2^, female: ≤ 34.29 cm^2^/m^2^), muscle strength (male: ≤ 22.32 kg, female: ≤ 16.39 kg), 6 m walking speed (≤ 0.89 m/s), SPPB score (≤ 8.67 points), PG-SGA score (> 3 points), and sarcopenia. The dependent variable was malnutrition in elderly patients with gastrointestinal tumors. A univariate logistic regression analysis was conducted, showing that the influencing factors for malnutrition in elderly patients with gastrointestinal tumors were L3-SMI (male: ≤ 41.29 cm^2^/m^2^; female: ≤ 34.29 cm^2^/m^2^), muscle strength (male: ≤ 22.32 kg; female: ≤ 16.39 kg), BMI (≤ 21.27 kg/cm^2^), SPPB score (≤ 8.67 points) PG-SGA score (> 3 points) and sarcopenia. (Table 2).

**Table 2:** Univariate logistic regression analysis of the influencing factors of malnutrition in elderly patients with gastrointestinal tumors.

Variable	β	SE	Wdlod χ^2^ value	OR (95%CI)	*p*
BMI (≤ 21.27 kg/cm^2^)	2.109	0.251	56.198	5.187 (2.187–11.287)	< 0.001
Tumor staging (stage II–III)	0.491	0.321	2.258	1.629 (0.871–3.062)	0.132
Calf circumference					
Male ≤ 30.01 cm	1.027	0.737	1.893	2.651 (0.781–10.372)	0.172
Female ≤ 29.37 cm	1.462	0.946	2.431	4.187 (0.831–18.918)	0.119
L3-SMI					
Male ≤ 41.29 cm^2^/m^2^	1.865	0.167	13.281	6.327 (2.187–8.176)	< 0.001
Female ≤ 34.29 cm^2^/m^2^	2.218	0.219	87.91	8.176 (2.187–13.298)	< 0.001
Muscle strength					
Male ≤ 22.32 kg	0.428	0.187	4.271	1.276 (1.009–2.321)	0.031
Female ≤ 16.39 kg	0.931	0.337	7.819	2.371 (1.271–4.387)	0.003
6 m walking speed (≤ 0.89 m/s)	0.619	0.357	3.009	1.876 (0.947–3.765)	0.086
SPPB score (≤ 8.67 points)	1.132	0.216	21.198	2.187 (1.276–4.281)	< 0.001
PG-SGA score (> 3 points)	0.887	0.261	10.761	2.217 (1.271–4.009)	< 0.001
Sarcopenia	0.583	0.182	10.482	1.746 (1.276–2.602)	< 0.001

*SE* standard error, *OR* odds ratio, *BMI* body mass index, *L3-SMI* third lumbar vertebra skeletal muscle index, *SPPB* short physical performance battery, *PG-SGA* patient-generated subjective global assessment.

### Multivariate logistic regression analysis of factors affecting malnutrition in elderly patients with gastrointestinal cancer

The independent variables were set as L3-SMI (male: ≤ 41.29 cm^2^/m^2^; female: ≤ 34.29 cm^2^/m^2^), muscle strength (male: ≤ 22.32 kg; female: ≤ 16.39 kg), BMI (≤ 21.27 kg/cm^2^), SPPB score (≤ 8.67 points), PG-SGA score (> 3 points), and sarcopenia. The dependent variable was malnutrition in elderly patients with gastrointestinal tumors. The multivariate logistic regression analysis revealed that the influencing factors of malnutrition in elderly patients with gastrointestinal tumors were BMI (≤ 21.27 kg/cm^2^) and sarcopenia, as laid out in Table 3.

**Table 3:** Multivariate logistic regression analysis of factors affecting malnutrition in elderly patients with gastrointestinal cancer.

Variable	β	SE	Wdlod χ^2^ value	OR (95%CI)	*p*
L3-SMI					
Male ≤ 41.29 cm^2^/m^2^	0.102	0.129	0.687	1.115 (0.876–1.387)	0.412
Female ≤ 34.29 cm^2^/m^2^	0.093	0.109	0.736	1.103 (0.881–1.378)	0.389
Muscle strength					
Male ≤ 22.32 kg	0.025	0.209	0.013	1.029 (0.701–1.539)	0.922
Female ≤ 16.39 kg	0.892	0.516	3.012	2.441 (1.021–6.761)	0.087
BMI(≤ 21.27 kg/cm^2^)	0.837	0.287	7.165	2.276 (1.261–3.287)	0.002
SPPB score (≤ 8.67 points)	0.251	0.213	1.376	1.289 (0.876–11.876)	0.242
PG-SGA score (> 3 points)	0.246	0.165	2.253	1.268 (0.917–1.765)	0.137
Sarcopenia	0.573	0.126	22.651	1.675 (1.376–2.176)	< 0.001

*SE* standard error, *OR* odds ratio, *BMI* body mass index, *L3-SMI* third lumbar vertebra skeletal muscle index, *SPPB* short physical performance battery, *PG-SGA* patient-generated subjective global assessment.

### Predictive ROC curve of sarcopenia in the diagnosis of malnutrition in elderly patients with gastrointestinal tumors

**Figure 1:**
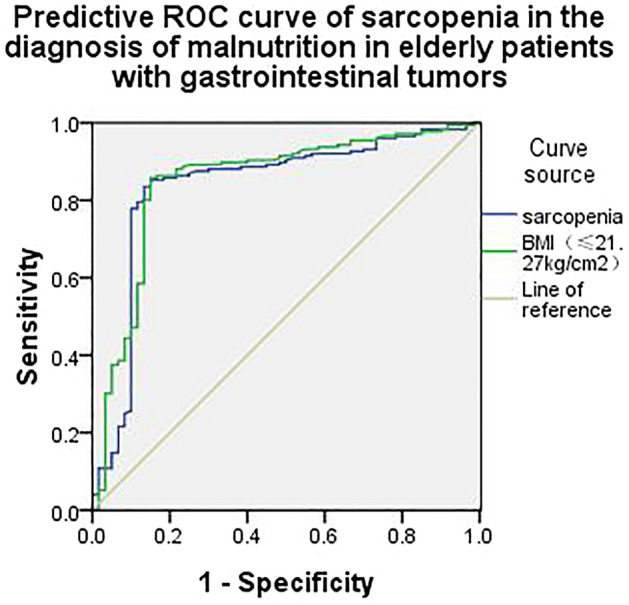
Predictive ROC curve of sarcopenia in the diagnosis of malnutrition in elderly patients with gastrointestinal tumors.

The ROC curve of BMI (≤ 21.27 kg/cm^2^) and sarcopenia, and the area under the curve (AUC) of BMI (≤ 21.27 kg/cm^2^) and sarcopenia predicting malnutrition in elderly gastrointestinal cancer patients were 0.681 and 0.881, respectively (Table 4 and Fig. 1).

**Table 4:** Values of predictive ROC curve of sarcopenia in the diagnosis of malnutrition in elderly patients with gastrointestinal tumors.

Variable	AUC	Cut-off value	Sensitivity (%)	Specificity (%)	*p*
BMI(≤ 21.27 kg/cm^2^)	0.681	22.32 kg	73.28	71.26	< 0.001
Sarcopenia	0.881		89.39	81.78	< 0.001

*BMI* body mass index, *AUC* area under curve, *ROC* receiver operating characteristic curve.

## Discussion

Gastrointestinal tumors are malignant tumors with high morbidity^14^. Recent statistics from the National Cancer Center indicate that gastric cancer ranks third in incidence, while colorectal cancer ranks fifth, posing a significant threat to the lives of residents^15^. Malnutrition is defined by the Experts Committee on Nutritional Therapy for Cancer Patients of Chinese Society of Clinical Oncology (CSCO) as a state where the lack or excess of energy, protein, and other nutrients adversely affects body function and clinical outcomes^16^. Tumor patients frequently suffer from malnutrition as a result of increased nutrient consumption, with about 40% to 80% of patients experiencing malnutrition and about 20% of tumor patients dying directly from malnutrition^17^. The specific location of gastrointestinal tumors exacerbates malnutrition. More than half of gastrointestinal tumor patients experience weight loss after diagnosis, and the causes of malnutrition in these patients are multifactorial^18^. Tumor cells can block the digestive tract when they proliferate, causing gastrointestinal disorders. Tumor necrosis factor α secreted by tumor cells can cause significant damage to the digestive tract, leading to inflammation. Nausea and vomiting after surgery can also affect food intake and absorption. Malignant tumors can result in metabolic abnormalities and weakened nutrient absorption. Finally, a lack of universal nutrition knowledge can lead to patients experiencing malnutrition or excess nutrition^18^. Previous research^19^ indicates that malnutrition significantly impacts the prognosis of gastrointestinal tumor patients. Muscular dystrophy, also known as sarcopenia, is defined by AWGS-2019 as a syndrome of age-related muscle mass loss accompanied by decreased muscle strength and physical function^20^.

Liu et al.^21^ showed that the incidence of malnutrition in patients with gastrointestinal tumors during recovery was 37.3%. Consistent with previous results, our study revealed that the incidence rate of malnutrition in elderly patients with gastrointestinal cancer was 45.21% (66/146). However, the studies supported by Fu et al.^22^ and Tan et al.^23^ showed that the respective incidence of malnutrition in patients with gastrointestinal/malignant tumors was 60.17% and 65%, which was inconsistent with and evidently higher than the results of this study. Reasonable nutritional support can play a positive role in the rehabilitation process of patients with nutritional deficiencies. It is necessary to select a simple, accurate, evidence-based medicine assessment of the nutritional status of elderly gastrointestinal cancer patients, not only to provide a strong guarantee for the rapid identification of patients requiring nutritional support, but also to offer objective theoretical basis for the appropriate nutritional support in clinical practice^24^. PG-SGA scale modified by Ottery is a reliable assessment tool for the nutritional status of cancer patients^10^. Therefore, this study applied the PG-SGA scale to assess the nutritional status of elderly gastrointestinal patients.

BMI, skeletal muscle index, and grip strength are components and functions used to assess nutritional status^25^. Qi et al.^26^ pointed out that the prevalence of sarcopenia in elderly patients with malignant tumors was 25.2%. Similarly, our study revealed the incidence rate of elderly gastrointestinal tumor with sarcopenia was 26.25% (21/80), with 25.00% (20/80) among those complicated with malnutrition, which was lower than 65% in the results by Qi et al.^27^. The main reason may due to the different criteria for diagnosing muscle failure. The results of this study demonstrated that, by comparing BMI, tumor staging, calf circumference, L3-SMI, muscle strength, 6 m walking speed, SPPB score, PG-SGA score, and sarcopenia (*p* < 0.05), malnutrition in elderly patients with gastrointestinal tumors was highly associated with the abovementioned variables.

Research by Zhang et al.^28^ depicted that BMI was a factor affecting malnutrition in elderly patients with gastrointestinal cancer. According to Ding et al.^29^, factors affecting malnutrition in patients with digestive system malignant tumors included BMI and pathological stage. Yu et al.^30^ showed that the calf circumference was closely related to the nutritional status of the elderly. Zhao et al.^31^ proposed that L3-SMI can be used to assess the nutritional risk of patients with advanced gastric cancer. Chen et al.^32^ asserted that the Sarcopenia Index can be used to diagnose malnutrition in patients with colorectal cancer. Feng et al.^33^ showed changes in muscle strength, skeletal muscle index, and 6-m walking speed in elderly patients with disuse muscular atrophy. Based on our study, the influencing factors for malnutrition in elderly patients with gastrointestinal tumors were L3-SMI (male: ≤ 41.29 cm^2^/m^2^; female: ≤ 34.29 cm^2^/m^2^), muscle strength (male: ≤ 22.32 kg; female: ≤ 16.39 kg), BMI (≤ 21.27 kg/cm^2^), SPPB score (≤ 8.67 points), PG-SGA score (> 3 points) and sarcopenia. Further multivariate logistic regression analysis showed that the influential factors for malnutrition in elderly patients with gastrointestinal tumors were BMI (≤ 21.27 kg/cm^2^) and sarcopenia. Yu et al.^34^ proposed that the evaluation of sarcopenia presented important clinical value in gastrointestinal tumors, with potential as an important tool to predict the clinical outcome of patients with gastrointestinal tumors. Meanwhile, patients with sarcopenia should seek early clinical intervention. According to Su et al.^35^, nutritional status was closely related to sarcopenia. Tang et al.^36^ suggested that body mass was an influential factor for malnutrition in patients with gastrointestinal cancer undergoing chemotherapy. In our study, BMI (≤ 21.27 kg/cm^2^) and sarcopenia had potential in predicting the occurrence of malnutrition in elderly patients with gastrointestinal tumors, which was consistent with previous research results.

## Conclusion

In conclusion, BMI (≤ 21.27 kg/cm^2^) and sarcopenia were significant factors influencing malnutrition in elderly patients with gastrointestinal tumors, which may predict the occurrence of malnutrition in such patients. However, considering the restricted sample size of our study, future studies with larger sample size were necessary to confirm the current findings.

## Data Availability

The datasets used and/or analysed during the current study are available from the corresponding author on reasonable request.
